# The evolution of contact calls in isolated and overlapping populations of two white-eye congeners in East Africa (Aves, *Zosterops*)

**DOI:** 10.1186/1471-2148-14-115

**Published:** 2014-06-02

**Authors:** Martin Husemann, Werner Ulrich, Jan Christian Habel

**Affiliations:** 1 Terrestrial Ecology Research Group, Department of Ecology and Ecosystem Management, Technische Universität München, Hans-Carl-von-Carlowitz-Platz 2, D-85354 Freising-Weihenstephan, Germany; 2 Institute of Biology/Zoology, Department of General Zoology, University of Halle, D-06120 Halle, Germany; 3 Nicolaus Copernicus University in Toruń, Chair of Ecology and Biogeography, Pl-87-100 Toruń, Poland

**Keywords:** Allopatry, Bioacoustics, Contact calls, Parapatry, Reproductive character displacement, Sympatry, *Zosterops abyssinicus*, *Zosterops poliogaster*

## Abstract

**Background:**

Closely related species often occur in geographic isolation, yet sometimes form contact zones with the potential to hybridize. Pre-zygotic barriers may prevent cross breeding in such contact zones. In East Africa, White-eye birds have evolved into various species, inhabiting different habitat types. *Zosterops poliogaster* is found in cool and moist cloud forests at higher elevations, whereas *Z. abyssinicus* is distributed across the dry and hot lowland savannahs. In most areas, these two species occur allopatrically, but in the contact zone where the mountain meets the savannah, the distributions of these species sometimes overlap (parapatry), and in a few areas the two taxa occur sympatrically. Acoustic communication is thought to be an important species recognition mechanism in birds and an effective prezygotic barrier for hybridisation. We recorded contact calls of both the lowland and highland species in (i) distinct populations (allopatry), (ii) along contact zones (parapatry), and (iii) in overlapping populations (sympatry) to test for species and population differentiation.

**Results:**

We found significant differences in call characteristics between the highland and lowland species, in addition to call differentiation within species. The highland *Z. poliogaster* shows a strong call differentiation among local populations, accompanied by comparatively low variability in their contact calls within populations (i.e. a small acoustic space). In contrast, calls of the lowland *Z. abyssinicus* are not differentiated among local sites but show relatively high variability in calls within single populations. Call patterns in both species show geographic clines in relation to latitude and longitude. Calls from parapatric populations from both species showed greater similarity to the other taxon in comparison to heterospecific populations found in allopatry. However, where the two species occur sympatrically, contact calls of both species are more distinct from each other than in either allopatric or parapatric populations.

**Conclusion:**

The contrasting patterns reflect divergent spatial distributions: the highland *Z. poliogaster* populations are highly disjunct, while *Z. abyssinicus* lowland populations are interconnected. Higher similarity in contact calls of heterospecific populations might be due to intermixing. In contrast, sympatric populations show reproductive character displacement which leads to strongly divergent call patterns.

## Background

Speciation and the maintenance of species boundaries are a major focus in evolutionary biology. The evolution of new species is continuously counteracted by the intermixing of individuals from adjoining populations [1]. Reproductive barriers can prevent the admixture of incipient species and are of major importance in closely related species that occur in close geographic proximity [2-4]. When species occur in sympatry, prezygotic mechanisms leading to the correct mate choice often evolve before any post-zygotic mechanisms (e.g. hybrid sterility) can be established [5-8]. Differences in signalling characters often initially evolve as a by-product of divergent ecological selection in allopatry [9,10]. When incipient species experience secondary contact, prezygotic isolation mechanisms can become enhanced as a result of selection against hybridization, a process termed reproductive character displacement [11-13]. This phenomenon has been documented for a variety of signalling characters, most commonly for acoustic communication. Examples come from a variety of organisms including grasshoppers, [14,15], crickets [16], amphibians [17,18], and birds [19-21].

The genus *Zosterops* is among the most species-rich bird genera worldwide and known for its high rates of microendemism and rapid speciation [22-25]. Geography and geological history have been identified as the main drivers of speciation in the genus [23]. Apart from differentiation driven by geographic isolation, intrinsic factors contribute to the species richness and often lead to a high level of endemism. For example, while many species of the genus are good dispersers [22], other species, especially narrow endemics, are characterized by ‘behavioural flightlessness’, the behavioural reluctance to disperse longer distances despite being able to fly, leading to differentiation [26,23,27]. In addition, representatives of the genus *Zosterops* seem to be morphologically and behaviourally dynamic. Rapid phenotypic change has been documented in *Zosterops* species and has been suggested to be an important feature of its greater potential for speciation [23,28]. If such rapid changes occur in reproductive characters, reproductive isolation can evolve quickly leading to speciation [29].

*Zosterops* is represented by various species across large parts of East Africa that inhabit a diverse range of habitats, from the dry lowland savannahs to high mountain cloud forests [30]. Where species have specific environmental niches, they generally do not overlap in their distributions. For example, winter temperatures limit the ranges of two sibling chickadee species in North America creating a dynamic hybrid zone [31]. Similar patterns have been observed in the *Heliconius* butterfly species-complex in South America [32]. Another example is represented by White-eye bird species. While the Mountain White-eye, *Zosterops poliogaster* requires moist and cool climatic conditions, its lowland congener, the Abyssinian White-eye *Zosterops abyssinicus* is found in the dry and warm lowland savannahs, which surround the mountains [30]. These opposite ecological demands cause that both species occur in allopatry. However, in some areas, these taxa occur in parapatry [30, JCH own observations]. In a few areas, the distributions of these species overlap such that they occur in sympatry [30]. However, little is known about their interactions (i.e. hybridization, competition) in such locations. This mosaic-like distribution pattern with allopatric, parapatric, and sympatric situations provides an excellent system to test for potential effects on the species’ behaviour and evolutionary processses. We hypothesize that reproductive character displacement will lead to stronger call divergence in sympatric populations compared to allopatric populations; populations with parapatric occurrences are assumed to represent some call similarities in the wake of intermixing along the contact zone. In detail we explore the following questions:

(i) Do the two bird species have distinct contact calls?

(ii) Are the two contrasting distribution patterns (disjunct versus connected) reflected in divergence patterns of contact calls?

(iii) Does parapatry with the congeneric species have an effect on call characteristics?

(iv) Does sympatry with the congener lead to enhanced differences of call characteristics?

## Results

### Differentiation between taxa

Contact calls of the two species were significantly differentiated from each other on the first two PC axes (ANOVA: PC1 DF = 1, F = 5446.22, p < 0.0001; PC2 DF = 1, F = 251.08, p < 0.0001). The first two principle components explained 93.3% of the total variance of the data (including both species). The highest and first peaks of calls had the highest loadings for PC1 (0.59 and 0.60 respectively) whereas the starting frequency had the highest loading for PC2 (Table 1) (0.80). Species differentiation was corroborated by Discriminant Function Analysis (Wilks’ Lambda = 0.436, F = 569.52, p < 0.0001); out of the 1808 *Z. abyssinicus* calls, 1615 were identified correctly (89%); for *Z. poliogaster* 1128 out of 1286 calls were identified correctly (88%). Interspecific comparisons of call similarity of populations of both species from parapatric sites showed that their contact calls were more similar to each other than the calls of both species from the allopatric and sympatric populations (ANOVA: F = 56.07, p < 0.001).

**Table 1:** Results from principal component analysis

**Variable**	**PC 1**	**PC 2**	**PC 3**
Starting frequency	0.285	0.358	0.258
First frequency peak	0.499	−0.259	0.000
Highest frequency peak	0.509	−0.245	0.010
End frequency	0.332	0.485	−0.173
Lowest frequency	0.350	0.538	−0.038
Total call length	−0.020	0.028	0.949
Range in frequency	0.421	−0.468	0.025
Eigenvalue	3.42	1.74	1.04
Explained variance	48.90	24.80	14.80

Shown are the loadings of the first three principle components explaining 88.5% of the total variance.

### Call patterns within species – differentiation

**Figure 1:**
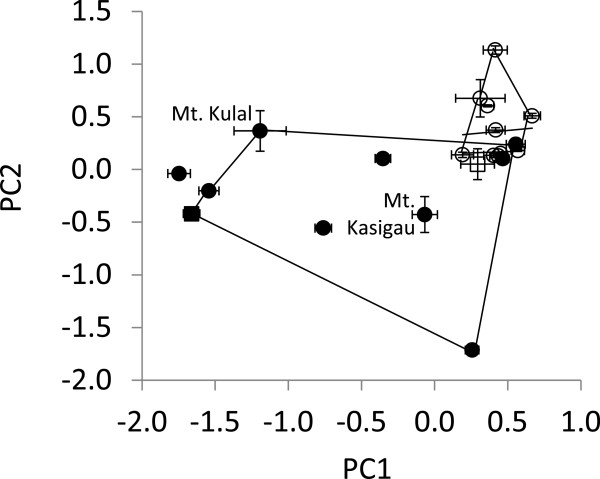
**Average positions (error bars denote two standard errors) of *Z. abyssinicus *** **(parapatric and allopatric - open circles, sympatric - open square) and *Z. poliogaster *** **populations (parapatric and allopatric - black circles, sympatric - black square) within two dimensional acoustic PCA space (strongly driven by a variation of starting and lowest frequencies and of frequency range in the highland species *Z. poliogaster *, for summary of loadings see Table **1**), convex hulls for both species exclude the respective sympatric populations.**

In both species two PC axes were identified explaining the major part of the variance (*Z. abyssinicus* PC1 = 38.8%, PC2 = 26.7%; *Z. poliogaster* PC1 = 52.4%, PC2 = 22.1%). The lowest frequency and the starting frequency had the highest loadings in *Z. abyssinicus*, whereas the first and highest peaks had the highest loadings in *Z. poliogaster* (Figure 1). ANOVA on the first two PC axes indicated that populations of both species are differentiated from each other (*Z. abyssinicus* DF = 11, F = 23.70, p < 0.0001; *Z. poliogaster* DF = 8, F = 596.23, p < 0.0001). Tukey tests showed that the population differentiation in *Z. abyssinicus* was strongly driven by the allopatric population at Hunters’ Lodge (PC1, most comparisons p < 0.0001, except for the allopatric population close to Mutito, p = 0.127). In *Z. poliogaster*, all populations were significantly differentiated for PC1 and PC2 (Figure 1). The highest intrapopulation variability was observed in the parapatric populations of Mt. Kasigau and Mt. Kulal.

**Figure 2:**
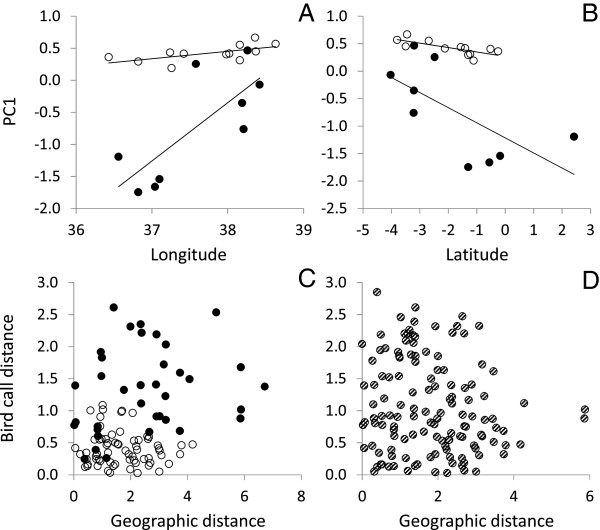
**Contact call variation along A) longitudinal and B) latitudinal gradients.** Black is the highland *Z. poliogaster*, white is the lowland *Z. abyssinicus*. **C)** Comparison of call distance (calculated as Euclidean distances from the first two principal coordinates axes for all pairs of sites) in response to geographic distance (degrees) for both species, **D)** respective interspecies comparisons. Regressions in **A**: *Z. poliogaster*: r^2^ = 0.59, p = 0.02; *Z. abyssinicus*: r^2^ = 0.38, p = 0.03. Regressions in **B**: *Z. poliogaster*: r^2^ = 0.44, p = 0.05; *Z. abyssinicus*: r^2^ = 0.56, p = 0.006.

For both species we found marked longitudinal (Figure 2A) and latitudinal (Figure 2B) trends in contact call patterns, while there was no clear distance decay in call pattern with geographic distance for either species (*Z. abyssinicus* Mantel r = −0.08, P > 0.5; *Z. poliogaster* Mantel r = 0.21, P > 0.1) or for the whole dataset (Figure 2C,D, Mantel r = 0.12, P > 0.1).

### Call patterns within species - acoustic space

**Figure 3:**
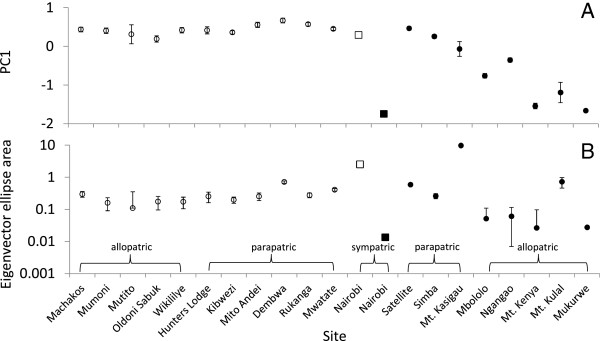
**Comparison of acoustic space as measured by first (dominant) eigenvector A. (upper panel) and the ellipse area B. (lower panel) spanned by the first two eigenvectors of principal coordinates analysis occupied by sym-, para-, and allopatric populations of the lowland *Zosterops abyssinicus *(white) and the highland *Z. poliogaster *(black).** The sympatric Nairobi populations are indicated by squares.

The lowland and highland species significantly differed in acoustic space, i.e. in the variance of contact calls among individuals and among distinct populations (Figure 3). While parapatric and allopatric populations of *Z abyssinicus* did not differ in contact calls, we found significant differences in *Z. poliogaster* (Figure 3). The eigenvector ellipse space of the highland *Z. poliogaster* is significantly larger (F-test: p < 0.001) than that of the lowland *Z. abyssinicus*, indicating the higher contact call variability among highland populations of *Z. poliogaster.* This difference is linked to greater variability of the starting and the lowest frequencies and the frequency range in the highland *Z. poliogaster* (Figure 3).

### Differentiation in sympatry

Nairobi is one location where both species can be found sympatrically. The acoustic spaces of the sympatric Nairobi populations of both species were outside the respective range of the conspecific populations as indicated by the convex hulls in Figure 1 and the eigenvector ellipse areas in Figure 3. Highest frequency and frequency range of the sympatric *Z. poliogaster* population of Nairobi differed significantly (t-test p < 0.01) from all other populations (Figure 1). Frequency range and total call length differentiated the sympatric Nairobi population of *Z. abyssinicus* from the other conspecific populations (t-test p < 0.001, Figure 1C). Both results show a shift of the sympatric Nairobi populations away from the typical call pattern of either species.

## Discussion and conclusion

Acoustic signal evolution is influenced by features of the physical habitat, community composition, ambient noise, phylogenetic history, morphological and physical constraints inherent to the species [13]. Our data suggest that several of these factors may be involved in the evolution of acoustic signalling in *Zosterops*. The two *Zosterops* species we studied have clearly distinct contact calls, mainly differing in the frequency range and the starting frequency of calls. Further, most populations within the two species are differentiated from each other in features of their contact calls. Differentiation was stronger in the isolated mountain populations of *Z. poliogaster* than in the panmictic lowland *Z. abyssinicus*. Call patterns in both species show a geographic cline suggesting similar effects of environmental selection. When considering the absence or presence of the congener, parapatric populations have relatively similar contact calls. In contrast, the sympatric populations of *Z. poliogaster* in the highland of Nairobi shows the strongest differentiation in contact calls from the lowland *Z. abyssinicus* suggesting reproductive character displacement. The sympatric *Z. abyssinicus* population also differed from all conspecific populations and shows a distinct shift away from *Z. poliogaster* with respect to total call length (Figure 1). In conclusion, it appears that both differences in local environmental conditions as well as selection due to co-existence with congenerics play an important role in the evolution of acoustic signalling in the genus. In the following we discuss these findings in detail.

### Species divergence

The two investigated species of *Zosterops* are clearly differentiated in their contact calls (Figure 1). Contact calls are considered an important, species-specific, social trait, as shown in previous studies for the genus *Zosterops*, suggesting a function for the maintenance of flock structure as well as for mate recognition [33,34]. Interestingly, the call patterns of some populations of *Z. poliogaster* strongly overlapped with *Z. abyssinicus*, but never in sympatry, indicating that divergent selection may be relaxed in non-overlapping populations.

### Population divergence

In addition to species differentiation, local populations of both species significantly differed in their calls, but the degree to which populations have diverged was different between the two species. In the lowland *Z. abyssinicus*, most populations showed little or no divergence in acoustic parameters (except for the population from Hunters Lodge, which was represented by a limited number of recordings, N = 78). In contras, local populations of the highland *Z. poliogaster* were much stronger differentiated (Figure 1). These different intraspecific patterns could be explained by the contrasting distributions of the two species: *Zosterops abyssinicus* is widely distributed in the lowland savannahs of East Africa with strong gene flow between local populations and very limited genetic differentiation [35] whereas *Z. poliogaster* populations are geographically isolated with little gene flow leading to strong genetic and morphologic differentiation [35,36]. The strong acoustic differentiation is also in line with the findings of Baker [37], who studied the bioacoustics in two isolated *Zosterops* populations and concluded that song patterns can evolve rapidly in this genus even across limited geographical space. However, whether the observed strong differentiation is the result of slight differences in local selective regimes or simply the product of drift cannot be inferred at this point.

In both species acoustic divergence follows geographic clines (Figure 2A,B), but not geographical distance between sites (Figure 2C,D). A correlation of longitude or latitude and acoustic parameters is a common finding in a variety of organisms (e.g. katydids, frogs and birds, [38-41]). In katydids such clinal variation in song patterns has been interpreted to be the result of hybridization and selection along the geographical gradient [41]. While we found clinal variation in both species, the differences among local populations in relation to longitude and latitude are more pronounced in the mountain species. This is likely due to lower connectivity and therefore less individual exchange among mountain populations as compared with the interconnected populations of the lowland taxon. Interestingly the clinal change is directed similarly in both species suggesting that similar environmental forces might be responsible. Several studies have focused on the traits influencing the rates of the evolution of syllable frequencies in different latitudes, altitudes and environments [42,43]. Analyses show that song frequency differences have evolved more rapidly at high latitudes, which may indicate an increased intensity of sexual selection [42,43].

### Differentiation in geographic proximity and sympatry

Besides the geographically isolated (allopatric) populations of both species, we recorded and analysed contact calls from three locations where the two species occur in parapatry (Mt. Kasigau, Taita Hills and Chyulu Hills). Our analyses indicate that calls of both species (highland and lowland taxa) are less different from each other than the average divergence found between both species. The call similarity in these parapatric populations might be the result of several processes: 1) if contact calls are a cultural trait and the species specific call patterns are learned rather than inherited, close proximity to a congener (but not complete overlap) might lead to the accidental admixture of acoustic signals among taxa during the imprinting phase, which has been shown for brood parasites [44]. Mixed singing due to heterospecific copying rather than introgression has also been shown in flycatchers [20]. 2) Alternatively, intermediate call patterns can be the result of occasional hybridization of two closely related species, if calls are genetically determined [45]. 3) A more recent study has suggested that interactions may occur more frequently between evolutionarily ‘old’ species; in such cases species interactions can drive phenotypic convergence across entire radiations [46]. In our case it is unknown whether contact calls are genetically fixed or learned. Furthermore, the evolutionary age of the species cannot be determined with certainty, therefore, it is difficult to distinguish between these alternatives. Phylogenetic analyses on the group are currently being undertaken and will allow testing of the latter hypothesis.

Our analyses suggest that when both species occur in sympatry (Nairobi population), the calls of each species show different deviations from the average call parameters. While for *Z. abyssinicus*, coexistence has relatively little effect on its contact call, *Z. poliogaster* exhibits a strong shift in call characteristics when compared to allo- and parapatric populations. The song pattern of the sympatric population does not overlap with the congener, and, represents the acoustically most distinct (conspecific) population. In addition, the acoustic space of the sympatric population is smaller when compared to all other *Z. poliogaster* highland populations or any population of *Z. abyssinicus*. The picture changes when populations are found in sympatry. Generally, sympatric species are rare in the genus *Zosterops,* which has led to the suggestion that species diversity is limited by competition with congeners [22]. In systems with multiple closely related species, interspecific competition for signal space [47] and selection against incorrect mate choice and hybridization [17,18] can lead to reproductive character displacement. Hence, if contact calls play a role in reproductive isolation in the system, it is expected that these species will have distinct calls when they occur sympatrically. This suggests that the coexistence with the congener leads to reproductive character displacement.

Character displacement is a common mechanism to reduce the risk of hybridization when closely related species occur in sympatry with examples from crickets [41], frogs [18,17] and birds [20,21]. Reproductive character displacement can either be symmetrical (meaning that both species diverge from their patterns exhibited in allopatry) or it can be asymmetrical (where only one species displaces) [21]. A study of sympatric populations of tinkerbirds for example showed a displacement of song patterns in both sympatric species [21]. In contrast, reproductive character displacement in sympatric populations of chorus frog species was asymmetrical [18]. In this example the rarer species displaced their calls, whereas the more common species at the location remained stable [18]. In our study, only *Z. poliogaster*, which likely has the smaller effective population size, showed character displacement, congruent with the previously described pattern of asymmetrical character displacement [18]. Our observation of reproductive character displacement suggests that contact calls might be under sexual selection in the genus *Zosterops* and could represent a prezygotic isolation barrier [48,14]. In addition, natural selection might further act to prevent hybridization because hybrids might be at a disadvantage as both species have different habitat preferences [31,32,49]. Therefore, there might also be ecological selection against hybrids.

Our study indicates distinct calls for the two White-eye species, with different intraspecific variances among local populations. These differences in their call variability reflect their contrasting spatial distribution, with strong disjunction in the highland *Z. poliogaster* populations and large and interconnected populations in *Z. abyssinicus*. Contact calls of heterospecific populations in parapatry are more similar to each other if compared with both, allopatric and sympatric populations. In contrast, when the species occur in sympatry, reproductive character displacement appears to occur leading to strongly divergent call patterns compared to divergence in calls of allopatric or parapatric populations.

## Methods

### Study species

Members of the family Zosteropidae are common models in evolutionary biology as they are known for their rapid diversification rates [22,23,50]. Little is known, however, about the mechanisms leading to such fast speciation. In this study we focus on different populations of the two East African *Zosterops* species, the highland *Z. poliogaster* and the lowland *Z. abyssinicus*. The Mountain White-eye, *Zosterops poliogaster* is restricted to moist and cool climatic conditions and thus occurs exclusively at higher elevations [51]. These specific environmental demands have led to long-term geographic isolation in distinct mountain massifs. In consequence, strong genetic and phenotypic differentiation is present, and has led to a debate on the current taxonomy of this group [30,35,52]. In contrast to these highly disjunct and differentiated populations of the Mountain White-eye, the lowland species *Z. abyssinicus,* the Abyssinian White-eye, is found in dry and warm lowland savannahs in large, interconnected populations [30]. Recent studies on this species showed a lack of intraspecific differentiation, and thus suggest strong panmixia among local populations [35]. It is not known how the high- and lowland species interact at contact zones.

### Studied populations

**Figure 4:**
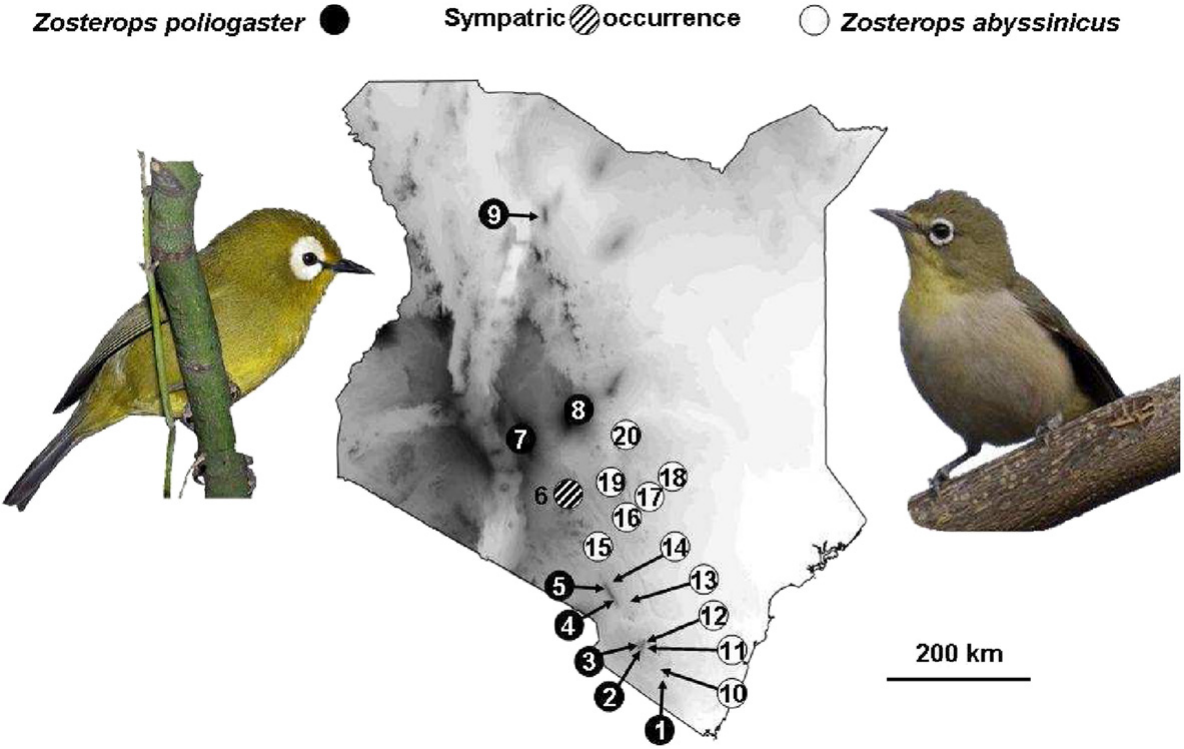
**Overview of all locations at which contact calls of the highland *Zosterops poliogaster *and the lowland *Z. abyssinicus *were recorded. **Site numbers coincide with Table 2.

We recorded contact calls of both the highland species *Z. poliogaster* and the lowland species *Z. abyssinicus* in areas where species occur (i) in allopatry, (ii) in parapatry (Mt. Kasigau, Taita Hills (two locations in the highland and two locations in the lowland), and Chyulu Hills (two locations in the highland and two locations in the lowland)), and (iii) in sympatry (the highlands of Nairobi). Calls were recorded at the following locations (from south to north): *Z. poliogaster* from Mt. Kasigau, Taita Hills (including two areas Ngangao, Mbololo, separated by a valley); Chyulu Hills (including the southernmost edge - Simba Valley, and the northernmost edge – Satellite), Nairobi, Mukurwe-ini, Mt. Kenya and Mt. Kulal; *Z. abyssinicus* was recorded from Rukanga (foothills of Mt. Kaisgau), Mwatate and Dembwa (both at the foothills of Taita Hills), Mtito Andei and Kibwezi (both in close geographic proximity to Chyulu Hills), Hunters Lodge, Machakos, Wikililye, Mutito, Odonio Sabuk, Mumoni Hills and Nairobi. Locations are displayed in Figure 4. An overview of all locations at which contact calls were recorded is provided in Table 2.

**Table 2:** **Overview of all populations of the highland *Zosterops poliogaster *** **and the lowland *Z. abyssinicus *** **used for bioacoustic analyses; given are running numbers (congruent with Figure **4**), name of each locality, number of contact calls analysed, date of recording and the coexistence type of each population**

**No**	**Locality**	N*	***N***	**Date**	**Type**
*Zosterops poliogaster*					
1	Mt. Kasigau	>50	73	02-2012	parapatric
2	Taita Hills-Ngangao	>50	190	02-2012	parapatric
3	Taita Hills-Mbololo	>50	172	02-2013	parapatric
4	Chyulu Hills - Simba valley	>100	248	02-2013	parapatric
5	Chyulu Hills - Satellite	>100	301	02-2012	parapatric
6	Nairobi	>50	205	02-2013	sympatric
7	Mukurwe-ini	>50	174	08-2013	allopatric
8	Mt. Kenya	>50	114	08-2013	allopatric
9	Mt. Kulal	>10	17	02-2010	allopatric
*Zosterops abyssinicus*					
10	Rukanga	>100	259	08-2013	parapatric
11	Mwatate	>150	359	02-2013	parapatric
12	Dembwa	>100	166	02-2013	parapatric
13	Mtito Andei	>50	129	02-2013	parapatric
14	Kibwezi	>100	301	02-2012	parapatric
15	Hunters Lodge	<50	78	02-2012	allopatric
16	Machakos	>50	160	08-2013	allopatric
17	Wikililye	>50	133	08-2013	allopatric
18	Mutito	>10	19	08-2013	allopatric
19	Oldonio Sabuk	>50	93	08-2013	allopatric
20	Mumoni Hills	>50	121	02-2013	allopatric
6	Nairobi	>20	41	02-2013	sympatric

*N based on estimates, taking into account possible multiple recordings of the same individual.

### Bioacoustic analyses

Contact calls were recorded during spring and summer 2010 and 2013 using a Sennheiser ME67 directional microphone (Sennheiser, Hanover, Germany). We selected the frequency curve 3 at the Sennheiser microphone to filter lower frequencies during recording. A digital Zoom-H4 recorder was used to save the calls as stereo wav-files. The input level was operated manually and adjusted to 100%. Contact calls of the birds were recorded with a distance of approximately five meters between the microphone and the target bird. The birds use contact calls to persist as flocks when they are moving through the thicket [34, JCH, pers. observation] and these calls may also function in mate recognition [33]. Calls are mostly emitted in series and regular intervals, often in parallel from various individuals. As both species occur in flocks (sizes ranging from a few individuals to some dozens), our data set may contain repeated recordings from same individuals. To limit repeated recording of the same individual (which would lead to non-independence of recordings), recording was stopped after a maximum of 5 clear and loud calls, and the next recording was performed at another edge of the bird flock. Thus we discriminate between the total number of sonograms analysed, and the estimated number of recorded individuals (see Table 2). Calls were recorded between 6:00 am and 6:00 pm for a period of three days per site.

**Figure 5:**
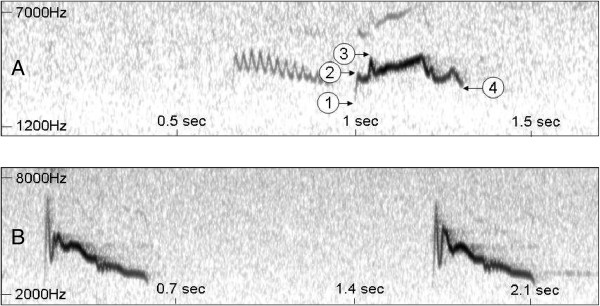
**Typical sonograms of the calls of the highland *Zosterops poliogaster *** **(A) and lowland *Zosterops abyssinicus *** **(B), displaying the parameters analysed for this study: 1: starting frequency (typically congruent with lowest frequency); 2: first peak (typically congruent with highest frequency); 3: highest frequency; 4: end-frequency (sometimes congruent with lowest frequency); additional characters measured were total call duration (seconds) and frequency range (highest to lowest frequency).**

Contact calls of high quality were further processed in the programme AUDACITY vers. 2.01 (Audacity Development Team). Calls affected by strong background noise or overlap with other calls were excluded from further analyses. After deleting calls of bad quality, a total number of 3353 calls remained (on average 160 calls per site ± 92, ranging from 17–359 calls per site). For each call we measured the following characters: starting frequency (sometimes congruent with lowest frequency), first peak (mostly congruent with highest frequency), end frequency (mostly congruent with lowest frequency), total duration of call (in seconds) and the range of frequency (difference between the lowest and the highest frequencies). Typical sonograms of both species displaying all analyzed characters are displayed in Figure 5. Spectral analyses were performed blind to site (i.e. population) and species using the programme PRAAT vers. 5.2.15 [53]. The Spectrogram settings menu was used to adjust the range of frequency (Hz) and the dynamic range (dB) depending on background noise.

### Statistics on species and population differentiation

We performed a Principal Component analysis (variance co-variance matrix with Z-transformed data: Z = (x-μ)/σ) to reduce data complexity. The first two PC axes explain 74% of the total variance of the data. Analysis of variance (ANOVA) was performed to test for species and population differences. First, we compared the two species. To further test if species had distinct calls we performed a discriminant function analysis. Two additional ANOVAs were performed to test for population differentiation within each species. Pairwise comparisons of conspecific populations were performed using Tukey’s HSD test.

### Statistics to test differentiation in sympatry

We calculated Euclidean distances between contact calls of all pairs of sites using the first two principal component axes (variance – covariance matrix) of the seven characters measured. We then compared these pairwise distances with the respective geographical distances to test if geographically proximate populations of the species differ more strongly in acoustic parameters than distant populations using Mantel correlation. To test for acoustic character displacement in sympatric populations we compared the average acoustic distances between both species (excluding the sympatric populations) with those at the sympatric Nairobi site.

We further estimated the acoustic space that allopatric and sympatric populations of each species occupy. For this all data were Z-transformed to meet assumptions of normality and homogeneity. We estimated the total acoustic space of all populations by generating two-dimensional convex hulls. Convex hulls of *Z. abyssinicus* and *Z. poliogaster* were calculated with the focal population included and excluded. We modified the approach of Jackson et al. [54] and calculated the eigenvector ellipse space spanned by the first two eigenvectors of principal coordinate analysis (Euclidean distances). This method captures a central tendency of acoustic space and is less biased by outliers than the convex hull method [44]. All statistical analyses were performed with Jmp vers. 10.0.0 (SAS Institute Inc.) and Past vers. 3.0 [55]. Our sample sizes are all well above (Table 2) the lower limit for unbiased results of ten data points identified by Jackson et al. [54].

## Competing interests

The authors declare that they have no competing interests.

## Authors’ contributions

JCH recorded contact calls and analysed the sonograms, MH and WU performed statistical analyses, MH wrote the first draft of the manuscript, all authors contributed in the interpretation of data and writing of the manuscript. All authors read and approved the final manuscript.
